# The ubiquitin ligase TRIM25 targets ERG for degradation in prostate cancer

**DOI:** 10.18632/oncotarget.11915

**Published:** 2016-09-08

**Authors:** Shan Wang, Rahul K. Kollipara, Caroline G. Humphries, Shi-Hong Ma, Ryan Hutchinson, Rui Li, Javed Siddiqui, Scott A. Tomlins, Ganesh V. Raj, Ralf Kittler

**Affiliations:** ^1^ Eugene McDermott Center for Human Growth and Development, University of Texas Southwestern Medical Center, Dallas, Texas, USA; ^2^ Department of Urology, University of Texas Southwestern Medical Center at Dallas, Dallas, Texas, USA; ^3^ Department of Pathology, Michigan Center for Translational Pathology, University of Michigan, Ann Arbor, Michigan, USA; ^4^ Simmons Comprehensive Cancer Center, University of Texas Southwestern Medical Center at Dallas, Dallas, Texas, USA; ^5^ Department of Pharmacology, University of Texas Southwestern Medical Center at Dallas, Dallas, Texas, USA; ^6^ Green Center for Reproductive Biology Sciences, University of Texas Southwestern Medical Center at Dallas, Dallas, Texas, USA

**Keywords:** prostate cancer, transcription factor, oncogene, ubiquitination

## Abstract

Ets related gene (ERG) is a transcription factor that is overexpressed in 40% of prostate tumors due to a gene fusion between *ERG* and *TMPRSS2*. Because ERG functions as a driver of prostate carcinogenesis, understanding the mechanisms that influence its turnover may provide new molecular handles to target the protein. Previously, we found that ERG undergoes ubiquitination and then is deubiquitinated by USP9X in prostate cancer cells to prevent its proteasomal degradation. Here, we identify Tripartite motif-containing protein 25 (TRIM25) as the E3 ubiquitin ligase that ubiquitinates the protein prior to its degradation. TRIM25 binds full-length ERG, and it also binds the N-terminally truncated variants of ERG that are expressed in tumors with TMPRSS2-ERG fusions. We demonstrate that TRIM25 polyubiquitinates ERG *in vitro* and that inactivation of TRIM25 resulted in reduced polyubiquitination and stabilization of ERG. TRIM25 mRNA and protein expression was increased in *ERG* rearrangement-positive prostate cancer specimens, and we provide evidence that ERG upregulates TRIM25 expression. Thus, overexpression of ERG in prostate cancer may cause an increase in TRIM25 activity, which is mitigated by the expression of the deubiquitinase USP9X, which is required to stabilize ERG.

## INTRODUCTION

Prostate cancer is the most common malignancy in men and the second leading cause of cancer-related death among men in the United States [1]. Fusions of E-twenty-six (Ets) transcription factor genes with androgen-responsive genes [2], are present in 50-70% of prostate-specific antigen (PSA) screened prostate cancers from populations of predominantly European descent. The most frequently rearranged member of this family is the *Ets Related Gene* (*ERG*), which is overexpressed through gene fusion with the 5′ untranslated region of the gene encoding *Transmembrane protease, serine 2 (TMPRSS2)* in ∼40% of prostate tumors [2, 3]. Depletion of ERG by RNAi decreases proliferation and/or invasiveness in prostate cancer cell lines [4, 5], and ectopic expression of ERG in aged mice [6] or in concert with loss of tumor suppressors in younger mice promotes prostate carcinogenesis [7-9]. Moreover, ERG controls a gene regulatory network related to the development of prostate cancer, and its progression to metastatic disease [10, 11].

The potential role of ERG as a cancer driver and the high incidence of the *TMPRSS2-ERG* gene fusion in prostate cancer have catapulted this protein into the forefront of new targets for therapeutic intervention. Like most transcription factors, ERG is considered an unconventional drug target due to the absence of an enzymatic activity or ligand-binding domain. Several strategies have been proposed for therapeutic targeting of ERG [12], such as by inhibiting its synthesis at the transcriptional or post-transcriptional level, or by blocking the interaction of ERG with its genomic targets, however, these strategies have not yielded yet pharmacological agents to test clinically in patients. We recently found that the deubiquitinase USP9X interacts with ERG in VCaP cells [13], which harbors the *TMPRSS2*-*ERG* gene fusion. Using this cell line, as well as other model systems in tissue culture and *in vivo,* we demonstrated that RNAi knockdown or inhibition of USP9X with the deubiquitinase inhibitor WP1130 efficiently ablates ERG, thus providing a new pharmacological avenue for targeting ERG by accelerating the turnover of the protein.

Our previous findings suggest that ERG stability in prostate cancer is modulated by the interplay of ubiquitination and deubiquitination. Importantly, two recent studies reported that full-length ERG protein is ubiquitinated in a Speckle-Type POZ Protein (SPOP)-dependent manner. Thus, low levels of ERG are maintained unless there are inactivating mutations in the SPOP E3 ubiquitin ligase adaptor protein [14, 15]. According to these studies, loss of the N-terminus of ERG when expressed from the *TMPRSS2*-*ERG* fusion gene results in increased stability of the truncated ERG protein due to loss of SPOP-dependent ubiquitination. Since we had previously demonstrated that N-terminally truncated ERG expressed from the *TMPRSS2*-*ERG* fusion gene is ubiquitinated and degraded (albeit truncated ERG appears to have a longer half-life than full-length ERG as reported in the two recent studies above), it is likely that there are SPOP-independent ubiquitin ligases that target ERG in fusion-positive prostate tumors. In the present study, we identified TRIM25 as an ubiquitin ligase that targets N-terminally truncated ERG in fusion-positive VCaP cells, and characterized its effects on ERG stability and its expression in prostate cancer.

## RESULTS

### Identification of TRIM25 as an ERG-interacting protein

**Figure 1:**
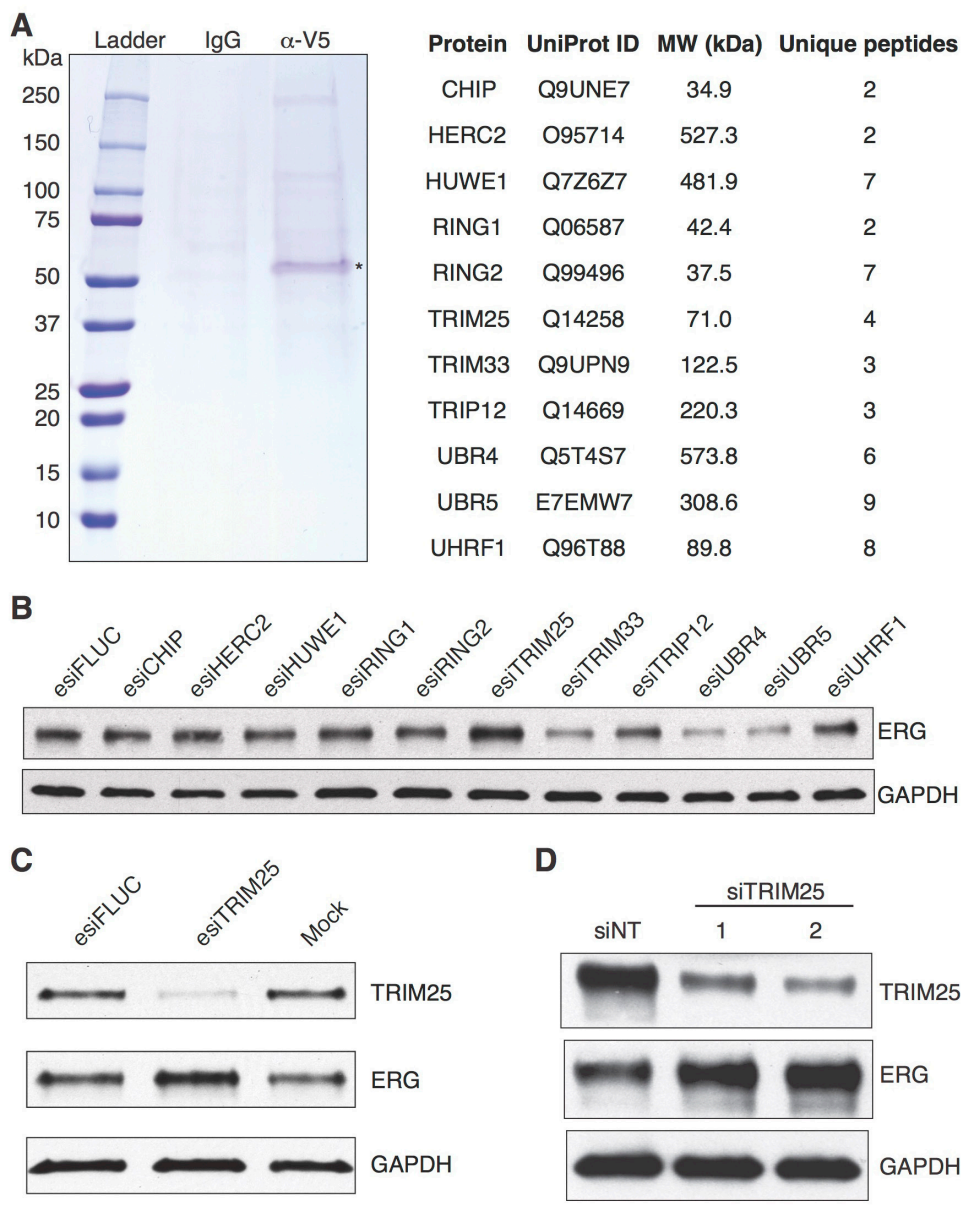
Identification of TRIM25 as a protein whose knockdown results in increased ERG protein levels **A.** Identification of proteins in VCaP whole cell extract that bind to ERG-V5. Eluted proteins were separated by SDS-PAGE and detected by Coomassie blue staining (left panel). The asterisk indicates the bait (ERG-V5). 11 putative ubiquitin ligases that were identified by mass spectrometry are listed (right panel). **B.**-**D.** TRIM25 knockdown increases ERG protein levels in VCaP cells. The 11 putative ERG-binding ubiquitin ligases were knocked down individually with endoribonuclease-prepared siRNAs (esiRNAs) in VCaP cells, and ERG protein levels were assayed 72 hours after knockdown **B.** Only TRIM25 knockdown resulted in increased ERG protein level. esiFLUC targets firefly luciferase and was used as negative control. We replicated the increased levels of ERG by TRIM25 knockdown with esiRNA **C.**, and validated this finding with two independent chemically synthesized siRNAs **D.**

Previously, we identified ERG interaction partners by performing co-immunoprecipitation studies of endogenous ERG in VCaP cells [13], but no ubiquitin ligases were identified that reduced ERG stability. Possible factors contributing to our inability to identify enzymes with the targeted activity include the following: 1) the interaction of such proteins with ERG may be transient in nature;2), the binding site of the ubiquitin ligase may be masked by the antibody used for immunoprecipitation; 3) the protein may be expressed at too low a level; 4) the immunoprecipitation may be too inefficient to detect the endogenous protein. To circumvent these issues we have now purified epitope-tagged recombinant ERG (ERG-V5) from HEK293T cells. The purified protein was used as bait for pulldown of ERG binding partners from whole cell extracts of VCaP cells. After elution of ERG-V5 and associated proteins, and separation by PAGE (Figure 1A), we identified putative ERG interacting proteins by mass spectrometry. Among these putative ERG-binding proteins were 11 ubiquitin ligases (Figure 1A, Supplementary Table 1). To determine which of these proteins were interacting nonspecifically and which were associated with regulation of ERG stability, we systematically knocked down each ubiquitin ligase transcript in VCaP cells using endoribonuclease-prepared siRNA (esiRNA) [16-18] and assessed the level of ERG protein levels. Only the knockdown of Tripartite motif-containing protein 25 (TRIM25) resulted in an increase in ERG protein levels (Figure 1B) as expected from a ubiquitin ligase that targets ERG for degradation.

We then examined the effect of inactivating TRIM25 using esiRNA, which efficiently reduced TRIM25 protein levels in VCaP cells (Figure 1C). We validated the specificity of the TRIM25 knockdown by testing two siRNAs that target independent regions of the TRIM25 transcript. Treatment of cells with both siRNAs resulted in efficient knockdown of TRIM25 and increase in levels of ERG (Figure 1D). Furthermore, we demonstrate that knockdown of TRIM25 with the same siRNAs causes increased protein levels of ERG in 22Rv1 prostate cancer cells (Supplementary Figure 1A) that express ERG-V5 from a stably integrated expression vector [13].

TRIM25 functions as an ubiquitin E3 ligase, and has been implicated in the regulation of innate immunity by mediating ubiquitination of RIG-I (Retinoic-acid-inducible gene-I) [19], a protein involved in interferon synthesis in response to viral infection. TRIM25 has also been shown to be regulated by estrogen receptor alpha in breast cancer and to mediate ubiquitination and degradation of two transcription factors: KLF5 [20] and ZFHX3 [21]. TRIM25 has not been implicated in prostate cancer biology, and no gain- or loss-of-function mutations for this gene have been reported for prostate cancer. We found that TRIM25 knockdown significantly increased migration of VCaP cells (Supplementary Figure 1B), which is inhibited by ERG knockdown [13]. Based on the identification of TRIM25 as an ERG-binding protein and on the observation that TRIM25 knockdown causes an increase in ERG protein levels in VCaP cells, we hypothesized that TRIM25 functions as an ERG ubiquitin ligase, which initiates a pathway leading to ERG degradation in fusion-positive prostate cancer.

### Mapping of interaction domains between ERG and TRIM25

Next we validated the physical interaction between ERG and TRIM25 by co-immunoprecipitating TRIM25 with endogenous ERG in VCaP cells (Supplementary Figure 1C). TRIM25 was also pulled down with GST-ERG from whole VCaP cell extract (Supplementary Figure 1D). Epitope-tagged ERG (ERG-HA) also co-immunoprecipitated with TRIM25-Flag (Supplementary Figure 1E).

**Figure 2:**
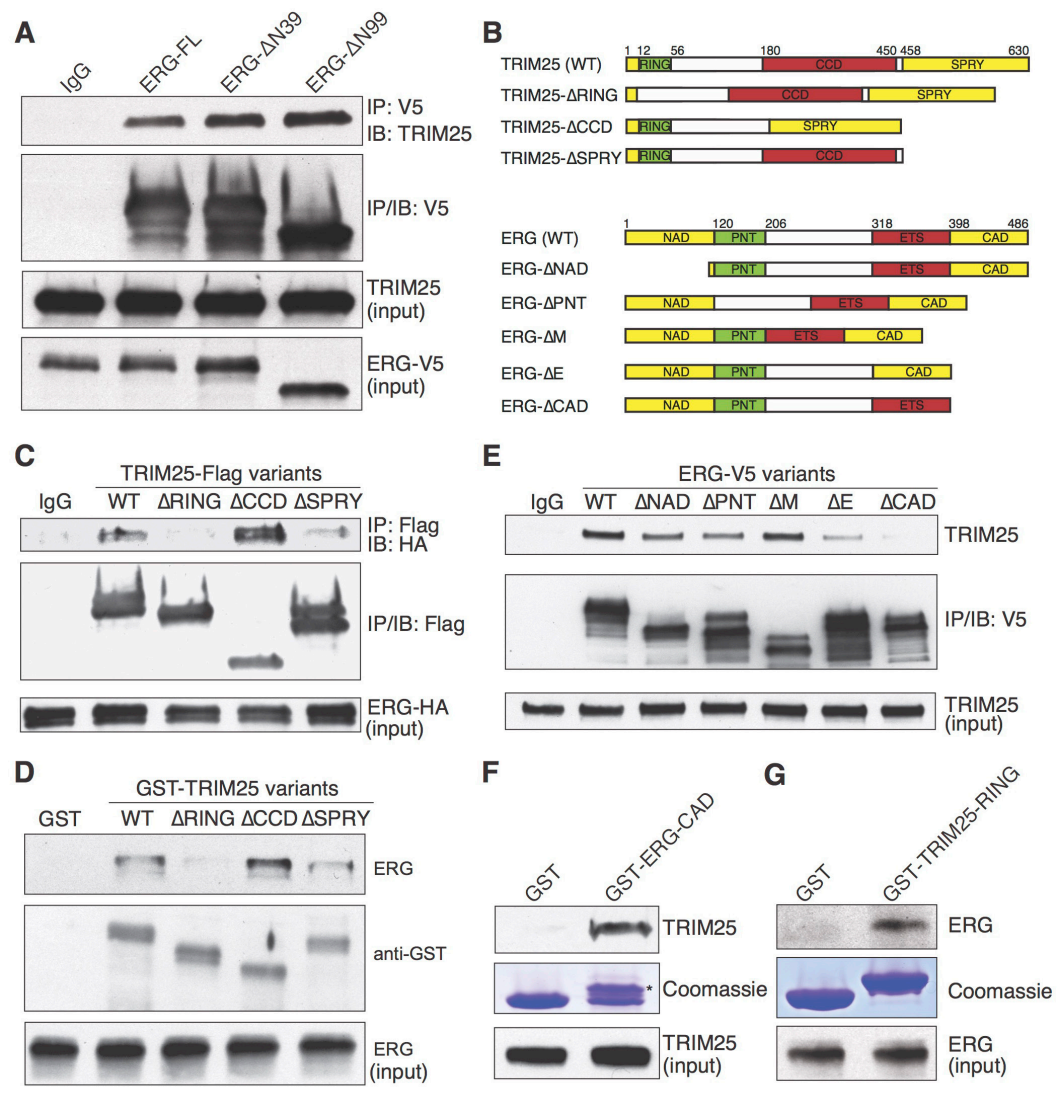
Analysis of TRIM25 interaction with ERG **A.** Co-immunoprecipitation of TRIM25 with full-length (ERG-FL) and N-terminally truncated ERG variants (ERG-ΔN39 and ERG-ΔN99). **B.**-**G.** Mapping of domains that are required for TRIM25 and ERG interaction. Epitope-tagged TRIM25 and ERG full-length and deletion mutants **B.** were used for co-immunoprecipitation and pulldown experiments. To identify critical TRIM25 domains **C.**, Flag-tagged TRIM25 variants and full-length HA-tagged ERG were ectopically expressed in HEK293T cells, then co-immunoprecipitation of ERG-HA was performed with an antibody against V5. Pulldown of endogenous ERG from VCaP cell extract with GST-TRIM25 variants (purified from *E. coli*) was used as an orthogonal method to validate the RING domain of TRIM25 as essential for ERG interaction **D.**. To identify critical ERG domains **E.**, V5-tagged ERG variants were ectopically expressed in HEK293T cells, then co-immunoprecipitation of endogenous TRIM25 was performed with an antibody against V5. Pulldown of endogenous TRIM25 or ERG from VCaP cell extract with the GST-tagged CAD-domain (*) of ERG **F.** or the RING domain of TRIM25 **G.**, respectively, was used to demonstrate that each of these domains is sufficient to mediate physical interaction between ERG and TRIM25. For all immunoprecipitation experiments the immunoblots shown for input represents 1% input.

The common TMPRSS2-ERG fusion proteins that lack 39 (ΔN39) or 99 (ΔN99) amino acids from the N-terminus of ERG were interacting with TRIM25, as we found that both truncated proteins co-immunoprecipitated with TRIM25 (Figure 2A). To identify the domains of ERG and TRIM25 that are required for their interaction, we performed co-immunoprecipitation experiments with deletion mutants expressed in HEK293T cells (Figure 2B). TRIM25 contains an N-terminal Really Interesting New Gene (RING) finger domain, a coiled-coil domain (CCD) and a C-terminal SPla and the RYanodine Receptor (SPRY) domain. The RING domain is essential for simultaneously recruiting ubiquitin-conjugating enzymes and substrates [22], the CCD domain may be essential for multimerization [23, 24], and the SPRY domain is required for substrate recruitment [23].

Flag-tagged TRIM25 deletion mutants lacking the RING domain did not co-immunoprecipitate HA-tagged ERG (Figure 2C), suggesting that this domain is critical for ERG recruitment. Deletion of the SPRY domain also reduced the amount of immunoprecipitated ERG. We validated these findings with a pulldown experiment, for which we expressed recombinant GST-tagged TRIM25 variants in E. coli for pulldown of ERG from whole VCaP cell extract (Figure 2D). We also performed co-immunoprecipitation experiments with V5-tagged ERG deletion mutants to identify ERG domains that are required for the interaction with TRIM25. Deletion of the C-terminal activation domain (CAD) of ERG (Figure 2E) nearly abolished co-immunoprecipitation of TRIM25, suggesting that this domain is essential for TRIM25 binding. Finally, we demonstrated in GST pulldown experiments that both the CAD domain of ERG and the RING domain of TRIM25 are sufficient to pull down TRIM25 (Figure 2F) or ERG (Figure 2G) from whole VCaP cell extract. These findings suggest that the RING domain of TRIM25 and the CAD domain of ERG are the most critical domains for mediating the physical interaction between these proteins.

### Analysis of TRIM25-mediated ubiquitination of ERG

**Figure 3:**
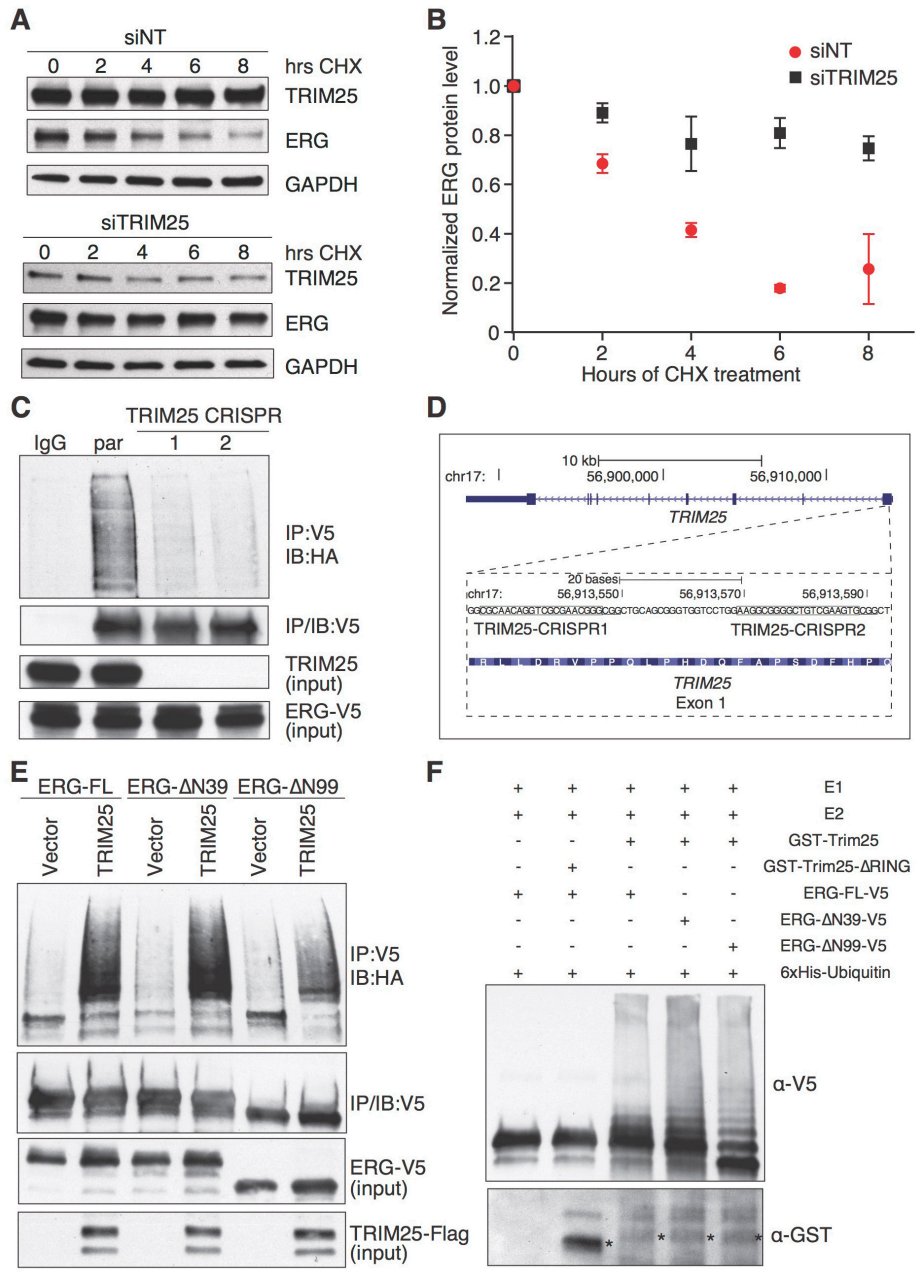
TRIM25 is a negative regulator of ERG stability and functions as an E3 ubiquitin ligase of ERG **A.**, **B.** TRIM25 knockdown in VCaP cells increases ERG stability in a cycloheximide (CHX) chase assay. Cells were treated over 8 hours with CHX (20 μg/ml) 72 hours after transfection of a siRNA targeting TRIM25 (siTRIM25) or a non-targeting control siRNA (siNT) **A.**. For densitometric analysis of immunoblots (*n* = 3, error bars represent s.e.m.) GAPDH was used for normalization **B.**. **C.**, **D.** TRIM25 is required for efficient ERG ubiquitination. ERG-V5 and HA-ubiquitin expression constructs were co-transfected into parental or *TRIM25* knockout HeLa cells, and after 24 hours ERG-V5 was immunoprecipitated with a V5 antibody and polyubiquitinated ERG was detected with an HA antibody **C.**. *TRIM25* knockout Hela cells were generated by CRISPR-Cas9-mediated gene editing using two guide RNAs that target independent sites (boxed, the PAM sequences are underlined) in the first exon of *TRIM25* as shown in the UCSC Genome Browser track **D.**. **E.** TRIM25 overexpression increases ubiquitination of full-length ERG as well as N-terminally truncated ERG-ΔN39 and ERG-ΔN99. HEK293T cells were transfected with expression vectors that drive expression of V5-tagged full-length ERG (ERG-FL), or the N-terminally truncated variants (ERG-ΔN39 and ERG-ΔN99), and expression vector for TRIM25-Flag (or empty vector as control) and HA-ubiquitin. **F.** GST-TRIM25 ubiquitinates V5-tagged full-length ERG, ERG-ΔN39 and ERG-ΔN99 *in vitro*. ERG-V5 variants were ectopically expressed in HEK293T cells, and immunoprecipitated for an *in vitro* ubiquitination assay using GST-TRIM25 (*) or GST-TRIM25-ΔRING (*), 6xHis-Ubiquitin, E1 and E2 conjugating enzymes. Immunoblotting was performed with V5 and GST antibodies. For all immunoprecipitation experiments the immunoblots shown for input represents 1% input.

The physical interaction of ERG with TRIM25 and the increased ERG protein levels after TRIM25 depletion suggest that TRIM25 may be an ERG-specific ubiquitin ligase and a major determinant of the rate of turnover of ERG. To test this hypothesis, we first analyzed the effect of TRIM25 inactivation on the stability of ERG in VCaP cells. We performed a time-course experiment examining the effect of knockdown of TRIM25 on ERG protein levels in cycloheximide-treated cells. Cells were transfected with siRNA against TRIM25 or a non-targeting control siRNA 72 hours before initiating cycloheximide treatment. TRIM25 depletion markedly increased the stability of ERG (Figure 3A, 3B).

Next, we tested the role of TRIM25 as a putative ERG-specific ubiquitin ligase using a cell-based ubiquitination assay. Because of low plasmid transfection efficiency and slow growth of VCaP cells, we analyzed the impact of TRIM25 loss on ubiquitination of ERG in HeLa cells (Figure 3C). We knocked out TRIM25 expression using CRISPR-Cas9 mediated gene editing (Figure 3D) [25]. Recombinant HA-ubiquitin and ERG-V5 were expressed in two independent HeLa cell knockout clones, as well as in parental HeLa cells and ERG-V5 was immunoprecipitated from cell lysates. The levels of ubiquitinated ERG-V5 was analyzed by immunoblotting using an HA antibody. Levels of polyubiquitinated ERG-V5 were markedly decreased in TRIM25 knockout cells. We verified the specificity of the ubiquitination assay by comparing V5 immunoprecipitates from HeLa cells expressing HA-ubiquitin only, or ERG-V5 in the presence or absence of HA-ubiquitin. In both cases, no ubiquitination signal was present in the HA immunoblot (Supplementary Figure 2A). Also, the V5 antibody markedly enriched ubiquitinated ERG-V5 as compared to IgG (Supplementary Figure 2B).

To further corroborate the role of TRIM25 as an ERG ubiquitin ligase we tested if TRIM25 overexpression increased ERG ubiquitination. For that purpose we expressed TRIM25-Flag or an empty vector together with full-length ERG-V5 and HA-ubiquitin in HEK293T cells. TRIM25-Flag expression markedly increased the ubiquitination of ERG-V5 (Figure 3E). We also examined the effect of TRIM25 overexpression on the ubiquitination of ERG-ΔN39-V5 and ERG-ΔN99-V5. We found that TRIM25-Flag overexpression markedly increased polyubiquitination of both N-terminally truncated variants of ERG, suggesting that TRIM25 mediates polyubiquitination of full-length, as well as truncated, ERG variants.

The decreased levels of polyubiquitinated ERG upon TRIM25 knockout and the increase in ERG ubiquitination upon overexpression of TRIM25 suggest that TRIM25 is an E3 ubiquitin ligase that targets ERG. To directly demonstrate this activity for TRIM25, we tested if ERG is ubiquitinated by TRIM25 *in vitro*. For this purpose, we incubated E1 and E2 conjugating enzymes, 6xHis-Ubiquitin and full length ERG-V5 (immunoprecipitated from HEK293T) with purified GST-tagged (expressed in *E. coli*) wildtype TRIM25 (GST-TRIM25) or a TRIM25 variant that lacked the RING domain. When incubated with GST-TRIM25 we observed ERG polyubiquitination, while no polyubiquitinated ERG-V5 was observed in the presence of the RING-domain deletion mutant of TRIM25 (Figure 3F). TRIM25 also polyubiquitinated ERG-ΔN39-V5 and ERG-ΔN99-V5. Collectively, these results establish that TRIM25 is an ubiquitin ligase of full-length ERG as well as the N-terminally truncated ERG variants that originate from the TMPRSS2-ERG gene fusion in prostate tumors.

Since TRIM25 was shown to mediate both K48- [21] and K63-linked [19] ubiquitination we sought to further characterize the mechanism of ERG ubiquitin chain linkage mediated by TRIM25. For this purpose, we used 6xHis-Ubiquitin K48 and 6xHis-Ubiquitin K63 (i.e., ubiquitin mutants where all lysine residues except K48 or K63 are substituted for arginine). We found an increase in polyubiquitinated ERG for both 6xHis-Ubiquitin K48 and 6xHis-Ubiquitin K63 (Supplementary Figure 2C). Since K48 chain linking generally targets proteins for proteasomal degradation, these results are consistent with the premise that TRIM25 promotes proteasomal degradation of ERG. In this experiment, we also found that the C-terminal activation domain of ERG was critical for ubiquitination *in vitro*.

### Analysis of TRIM25 expression in prostate cancer

**Figure 4:**
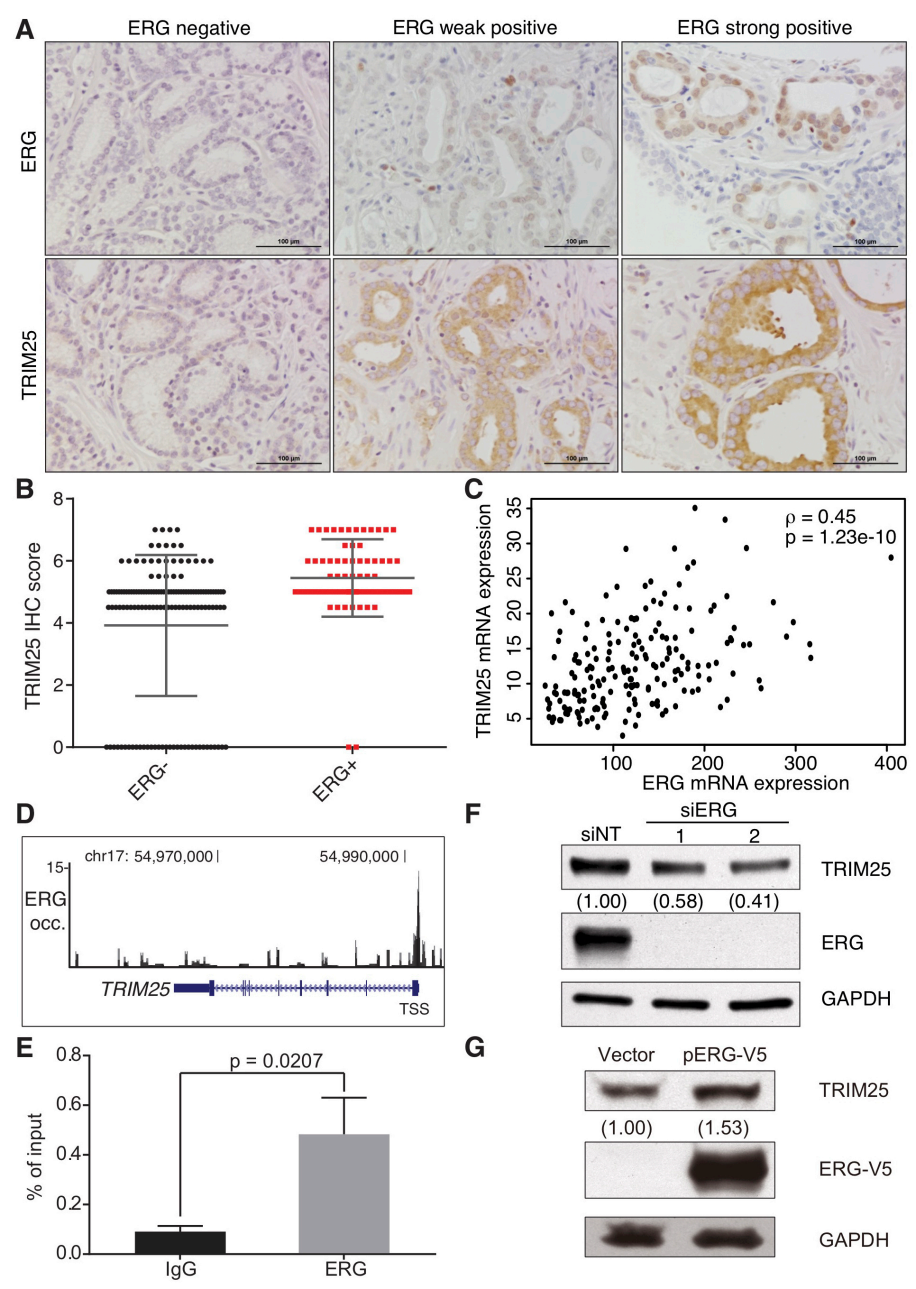
ERG is a positive transcriptional regulator of TRIM25 expression in prostate cancer **A.**, **B.** Analysis of TRIM25 and ERG protein expression by immunohistochemical (IHC) analysis in a tissue microarray of 209 prostate cancer specimens. Slides were stained for TRIM25, ERG and hematoxylin, and representative images are shown for specimens with variable staining intensities for ERG and TRIM25 **A.**. Scale bars are 100 μm. TRIM25 protein levels are significantly higher in the 70 ERG-positive samples than in the 139 ERG-negative samples (*p* = 3.89e-07, Student's *t*-test) **B.**. **C.** Paired TRIM25 and ERG mRNA expression of 193 ERG-positive prostate cancer samples from TCGA (ρ - Spearman's correlation coefficient). **D.**, **E.** ERG occupancy is markedly increased at the transcription start site (and putative promoter region) of *TRIM25*, as depicted in the UCSC Genome Browser for ERG ChIP-seq data **D.**. The y axis depicts ChIP-seq read density, which reflects ERG occupancy. We validated the ChIP-seq data experimentally by ChIP-qPCR analysis of ERG occupancy at the TRIM25 promoter in VCaP cells **E.** (*n* = 3, *p* value - *t* test, error bars represent s.d.). **F.** ERG knockdown in VCaP cells results in reduced TRIM25 expression. Cells were transfected with siRNAs against ERG or a non-targeting control siRNA (siNT), and immunoblotting for ERG, TRIM25, and GAPDH was performed 72 hours after transfection. **G.** Ectopic expression of ERG-V5 in 22Rv1 cells results in increased TRIM25 expression. 22Rv1 cells were transfected with an ERG-V5 expression vector or the empty vector, and selected for stable expression with geneticin. Cells were analyzed by immunoblot analysis for TRIM25, ERG-V5, and GAPDH expression. For densitometric analysis of immunoblots GAPDH was used for normalization.

We examined the expression of TRIM25 protein by immunohistochemical analysis (Figure 4A) in 209 prostate cancer tumor samples that were placed on a tissue microarray generated at the University of Michigan. Significantly higher levels of TRIM25 were present in the tumors that expressed ERG (*p* = 3.89e-07) than in the ERG-negative tumors (Figure 4B). This observation may appear to be counterintuitive because high TRIM25 protein expression would be expected to result in lower ERG protein levels. However, we note that the deubiquitinase USP9X, which removes ubiquitin from ERG, is also expressed at higher levels in ERG-positive tumors and correlates with the levels of ERG protein in these tumors [13]. Furthermore, we demonstrate here that catalytically active USP9X (but not catalytically inactive USP9X in which serine was substituted for cysteine at position 1566 [13]) reverts TRIM25-mediated ubiquitination of ERG *in vitro* (Supplementary Figure 2D). Thus, the expression of TRIM25 in prostate cancer cells may not result in reduced ERG protein levels when USP9X is also expressed.

Surprisingly, we found a positive correlation between the transcript levels of TRIM25 and ERG in ERG-positive prostate tumors (Figure 4C) in the RNA-seq data from The Cancer Genome Atlas (TCGA) consortium. This positive correlation between ERG and TRIM25 mRNA expression suggests that the transcription factor ERG may regulate TRIM25 mRNA expression. Several lines of evidence support this hypothesis: First, we found high ERG occupancy in the vicinity of the transcription start site of TRIM25 when we analyzed ChIP-seq data generated for ERG in VCaP cells (Figure 4D). We validated the enrichment of ERG at the *TRIM25* gene promoter in VCaP cells with a chromatin immunoprecipitation and quantitative PCR assay, which demontrated 5.3-fold greater enrichment for ERG occupancy (Figure 4E). Thus, ERG appears to be bound to the *TRIM25* gene promoter. We found that ERG knockdown with two independent siRNAs resulted in 42% or 58% reduction of TRIM25 expression in VCaP cells (Figure 4F). And, when we analyzed the impact of ectopic ERG expression in 22Rv1 cells that do not harbor any Ets gene fusions we found that ERG-V5 expression increased TRIM25 expression by 53% (Figure 4G). These results suggest that ERG may positively regulate TRIM25 expression, which could present a negative feedback mechanism to maintain physiological ERG protein levels. Our previous discovery of USP9X as an ERG-stabilizing deubiquitinase [13] suggests that reduction of ERG protein levels by TRIM25-mediated proteasomal degradation is prevented by expression of USP9X in fusion-positive prostate cancer cells.

## DISCUSSION

We have identified TRIM25 as an ERG-binding ubiquitin ligase in prostate cancer cells. We demonstrated that TRIM25 knockdown results in increased ERG stability in *TMPRSS2-ERG* expressing prostate cancer cells. Using several biochemical assays we show that TRIM25 mediates the polyubiquitination of full-length ERG as well as N-terminally truncated ERG. In conclusion, our work provides novel insights in the regulation of ERG protein stability in prostate cancer.

## MATERIALS AND METHODS

### Identification of ERG interacting proteins

ERG-V5 was ectopically expressed in HEK293T cells, immunoprecipitated with a V5 antibody, and used as bait for pulldown of ERG-binding proteins from VCaP cell lysates. Eluted proteins were separated by SDS-PAGE. For Mass spectrometry, PAGE gel slices were digested with trypsin, and high performance liquid chromatography tandem mass spectrometry (HPLC/MS/MS) analysis of tryptic peptides was performed with a Thermo Fusion Lumos mass spectrometer (Thermo) coupled to an Ultimate 3000 RSLC-Nano liquid chromatography systems (Dionex).

### Co-immunoprecipitation

Full-length TRIM25-FLAG, ERG-V5, ERG-HA, deletion variants of ERG-V5 and TRIM25-Flag, and/or HA-ubiquitin expression plasmids were transfected into HEK293T or HeLa cells, and immunoprecipitation was performed with V5, FLAG or HA antibodies.

### Protein purification and GST Pulldown

GST-TRIM25 and GST-TRIM25-ΔRING were expressed in *E. coli* BL21(DE3), and purified with glutathione sepharose beads and glutathione elution. For GST pulldown, whole VCaP cell extracts were incubated with GST or GST-TRIM25 variants, and eluted proteins were detected by immunoblot analysis.

### Analysis of ERG protein stability in VCaP cells

VCaP cells transfected with siRNA against TRIM25 or a non-targeting control were treated with cycloheximide, or DMSO. Immunoblot analysis of TRIM25, ERG, and GAPDH expression was performed for several time points over 8 hours.

### Generation of *TRIM25*-knockout HeLa cells

We used the GeneArt^®^ CRISPR Nuclease (OFP Reporter) Vector Kit (Thermo Fisher Scientific) according to the manufacturer's instructions to generate *TRIM25* CRISPR nuclease constructs, which were transfected into HeLa cells with Effectene transfection reagent (Qiagen). *TRIM25*-knockout HeLa cells clones were identified by immunoblot analysis.

### Analysis of ERG ubiquitination in *TRIM25*-knockout HeLa cells

*TRIM25*-knockout HeLa cells grown in DMEM with 10% FBS were co-transfected with ERG-V5 and HA-ubiquitin expression plasmids. Immunoprecipitation with a V5 antibody was performed as described above. For Western blot analysis antibodies against TRIM25, HA, V5, and GAPDH were used.

### *In vitro* ubiquitination assay

ERG-V5, ERG-ΔN39-V5 and ERG-ΔN99-V5, was overexpressed in HEK293T cells and immunoprecipitated. These purified proteins were incubated with recombinant GST-TRIM25 variants purified from *E. coli*, human recombinant UBE1, UbcH5a and 6xHis-Ubiquitin (all three proteins were purchased from Boston Biochem).

### Immunohistochemical staining

Immunohistochemical staining was performed as previously described (Strand, 2012; Ma, 2010) for ERG (1:100, Biocare Medical) and TRIM25 (1:100, Abcam). ERG and TRIM25 expression were evaluated on a previously described tissue microarray containing 209 cases of localized prostate cancer arrayed in triplicate [26]. All patients underwent radical prostatectomy at the University of Michigan Health System as primary monotherapy without neoadjuvant hormonal or radiation therapy. This radical prostatectomy series is part of the University of Michigan Prostate Cancer Specialized Program of Research Excellence Tissue Core. Tumor cells with ERG and/or TRIM25 staining were scored manually per tissue core by a reviewer who was blinded to the clinical data.

## SUPPLEMENTARY MATERIAL TABLES AND FIGURES


