# Supplementary material for: The ubiquitin ligase TRIM25 targets ERG for degradation in prostate cancer
Source: Oncotarget. 2016 Sep 8;7(40):64921–31. doi: 10.18632/oncotarget.11915 (PMC5323126; doi:10.18632/oncotarget.11915)
Supplement: Supplementary file 1 [file oncotarget-07-64921-s001.pdf]

# The ubiquitin ligase TRIM25 targets ERG for degradation in prostate cancer

**Supplementary Material**

**Plasmids.** cDNA fragments containing the reading frames of ERG and TRIM25 were amplified by PCR using primers appended with restriction site sequences and cloned into the pCMV-TnT vector (Promega) or pIRESneo3 (Promega) that had been modified with a C-terminal V5, HA or 3xFlag tag or into the pGEX6P-2 vector (GE Healthcare Life Sciences). ERG-ΔN39, ERG-ΔN99, ERG-ΔNAD, ERG-ΔPNT, ERG-ΔM, ERG-ΔETS, ERG-ΔCAD, TRIM25-ΔRING, TRIM25-ΔCCD and TRIM25-ΔSPRY were subcloned into pCMV-TNT vector with V5 or 3xFLAG tag. pRK5-HA-Ubiquitin was obtained from Addgene. All primer sequences are listed in **Supplementary Table 2**.

**RNAi reagents.** Endoribonuclease-prepared siRNAs (esiRNA) were prepared as previously described (Kittler et al., 2005; Kittler et al., 2004; Kittler et al., 2007). In brief, esiRNA target regions that were predicted *in silico* to produce the most specific and efficient esiRNAs were amplified from a human cDNA library with primers appended with T7 RNA polymerase promoter sequence. The PCR products were used for *in vitro* transcription of RNA with T7 polymerase, followed by denaturation and annealing to generate long double-stranded RNA, which was digested with GST-RNase III to esiRNAs of 19-25 bp, which were then purified by ion exchange chromatography and isopropanol precipitation. Chemically synthesized ON-TARGET *plus* siRNAs were obtained from Dharmacon. All esiRNA primer sequences and siRNA sequences are listed in **Supplementary Table 2**.

**Cell culture and transfection.** VCaP cells, HeLa cells, 22Rv1 cells and HEK293T cells were obtained from ATCC and cultured in DMEM with 10% FBS. Provenance of all cell lines was validated prior to submission by microsatellite fingerprinting in the McDermott Sequencing Core (UT Southwestern). Plasmids were transfected with Effectene Transfection Reagent (Qiagen); for siRNA transfection Lipofectamine RNAiMax (Invitrogen) was used for HeLa and HEK293T cells, HiPerFect Transfection Reagent (Qiagen) and Stemfect RNA Transfection Reagent (Stemgent) were used for VCaP cells. To generate cells that stably express ERG, 22Rv1 cells were transfected with pIRESneo3-ERG-V5 plasmids or pIRESneo3 (empty vector) and cells stably expressing the IRES-neo transcripts were selected with geneticin (600 μg/ml).

**Antibodies and reagents**. The following antibodies were used for immunoprecipitation: ERG (Epitomics, 5115) or (Abcam, ab133264 for chromatin IP), IgG (Cell Signaling, 2729), V5 (Genscript, A01724) and HA (Santa Cruz Biotechnology, sc-7392); Immunoblot analysis: TRIM25 (Abcam, ab167154), HA (Bethyl Laboratories, A190-108A, or Sigma, H3663, or Cell Signaling Technology, 3724), V5 (Bethyl Laboratories, A190-120A), ERG (Epitomics, 2805, or Santa Cruz, sc-271048), GAPDH (Cell Signaling Technology, 2118), Flag M2 (Sigma, F3165) and β-actin (Cell Signaling Technology, 4970); Immunohistochemistry: TRIM25 (Abcam, ab167154), ERG (Biocare Medical, CM421). The following reagents were used: cycloheximide (Sigma); Ubiquitin Activating Enzyme (UBE1) (BostonBiochem, E-305), UbcH5a/UBE2D1 (BostonBiochem, E-616), HA-Ubiquitin (BostonBiochem, U-110), 6xHis-Ubiquitin (BostonBiochem, U-530), 6xHis-Ubiquitin K48 (BostonBiochem, UM-HK480) and 6xHis-Ubiquitin K63 (BostonBiochem, UM-HK630), V5 peptide (Sigma, V7754).

**ERG immunoprecipitation and Mass spectrometry.** ERG-V5 was transiently expressed in HEK293T cells by transfection with the pCMV-ERG-V5 expression vector. 48 hours after transfection, cells were lysed (lysis buffer: 25mM HEPES [pH7.5], 400 mM NaCl, 0.5% IGEPAL CA-630, 1mM DTT, 5% glycerol and protease inhibitors). The soluble fraction of the lysate was diluted to adjust the NaCl concentration and IGEPAL CA-630 concentration to 100 mM and 0.125%, respectively, and then pre-cleared with GammaBind G Sepharose (GE Healthcare). Pre-cleared lysates were incubated with a mouse anti-V5 antibody (Genscript, A01724) at 4°C for 3 hours, followed by immunoprecipitation with GammaBind G Sepharose beads at 4°C for 1 hour. Then beads were washed four times with 100 mM NaCl and 0.1% IGEPAL CA-630. Proteins were eluted with V5 peptide (500 ng/ml) at 4°C for 30 minutes. The eluted fraction was mixed with 2X Laemmli buffer and boiled for subsequent SDS-PAGE. For mass spectrometry, PAGE gel slices with bands of interest were digested with trypsin and desalted with C18 ZipTips (Millipore) according to the manufacturer's instructions. An integrated system that includes an Agilent 1100 series Nanoflow LC system (Agilent) and a LTQ 2D trap mass spectrometer (Thermo Electron) equipped with a nanoelectrospray ionization (NSI) source was used to perform high performance liquid chromatography tandem mass spectrometry (HPLC/MS/MS) analysis: Tryptic peptides were separated by a capillary HPLC column packed in-house with Luna C18 resin (Phenomenex). The eluted peptides were directly electrosprayed into the LTQ ion trap mass spectrometer, with a data-dependent mode. Mascot (version 2.3, Matrix Science) database was used to map peptides to proteins.

**Migration assay.** For the migration assay, VCaP cells were transfected with TRIM25 siRNA (#1) or ERG siRNA (#1) or non-targeting siRNA for 48 hours. Cells were then reseeded on the

outer surface of an 8.0-mm 24-well plate Transwell® insert (Corning Costar) with serum-free medium and complete medium in the lower chamber. After 24 hours of incubation at 37 °C with 5% $CO_2$, cells remaining on the outer surface of the Transwell insert were gently removed with a cotton swab. Migrated cells (adherent to the inner surface of the insert) were stained with 1.0% crystal violet and air-dried. To quantify the migration, inserts were incubated with 200 µl of 10% acetic acid (v/v), the OD at 560 nm of the resulting solution was measured with a spectrophotometer (BMG Labtech).

**Protein purification and GST Pulldown**. GST-TRIM25 protein and GST-TRIM25-ΔRING were expressed in *E. coli* BL21(DE3) after transformation of the pGEX6P-2 expression vector. After induction for 5 hours with 0.4 mM IPTG at 32°C, bacteria were pelleted by centrifugation. The pellets were resuspended in lysis buffer (10 mM $Na_2HPO_4$, 1.8 mM $KH_2PO_4$, [pH 7.4], 300 mM NaCl, 2.7 mM KCl, 0.5% Triton X-100, 5% glycerol, 1 mg/ml Lysozyme, 1 mM PMSF, 2 mM DTT). After incubating the resuspended cells on ice for 1 hour, lysis was performed by sonication, and the lysate was cleared by centrifugation. Glutathione sepharose 4B beads (GE Healthcare) were added to the supernatant and incubated with gentle agitation at 4°C for 2 hours. The beads were washed four times with lysis buffer. Then, GST-fusion proteins were eluted with 10 mM glutathione and stored at -80°C. For the GST pulldown, VCaP cells were lysed in high salt buffer (20 mM Tris [pH 8.0], 1.5 mM $MgCl_2$, 0.2 mM EDTA, 0.5 mM DTT, 400 mM NaCl, 0.5% IGEPAL CA-630, Protease Inhibitor Cocktail [Sigma]). Extracts were diluted to adjust the NaCl concentration to 100 mM and IGEPAL CA-630 to 0.125%, and subjected to pre-clearing with GST beads. Pre-cleared supernatants were incubated with 30 µl GST or GST-fusion protein beads for 2 hours at 4°C. The beads were then washed with washing buffer (20 mM Tris [pH

8.0], 1.5 mM $MgCl_2$, 0.2 mM EDTA, 0.5 mM DTT, 100 mM NaCl, 0.1% IGEPAL CA-630) four times, boiled in 2X Laemmli buffer for SDS-PAGE. Eluted proteins were separated on a 4-20% SDS-PAGE gel and visualized by Coomassie blue staining or detected by immunoblotting.

**Co-immunoprecipitation.** TRIM25-FLAG, ERG-V5, ERG-HA, truncated ERG-V5 and/or HA-ubiquitin expression plasmids were transfected into HEK293T cells. Twenty-four hours after transfection cells were lysed in lysis buffer (25mM HEPES [pH7.5], 400 mM NaCl, 0.5% IGEPAL CA-630, 1mM DTT, 5% glycerol and protease inhibitors). The soluble fraction of the lysate was diluted to adjust NaCl concentration and IGEPAL CA-630 concentration to 100 mM and 0.125%, respectively, and then pre-cleared with GammaBind G Sepharose (GE Healthcare). Pre-cleared lysates were incubated with V5, FLAG or HA antibodies at 4°C for 3 hours, followed by immunoprecipitation to the sepharose beads at 4°C for 1 hour. Beads were washed four times with 100 mM NaCl and 0.1% IGEPAL CA-630, boiled in Laemmli buffer for Western blot analysis.

**Construction of the *TRIM25* CRISPR/Cas9 vector**. To generate *TRIM25*-knockout HeLa cells, the *TRIM25* gene was edited using the GeneArt® CRISPR Nuclease (OFP Reporter) Vector Kit (A21174, Thermo Fisher Scientific) according to the manufacturer's instructions. Briefly, *TRIM25* sgRNA-specific oligonucleotides for (synthesized by Sigma, sequences are listed in Table S2) were annealed to form a double-stranded oligonucleotide with compatible ends for cloning and ligated into the GeneArt® CRISPR Nuclease (OFP Reporter) Vector. The constructs were introduced into NEB® 5-alpha Competent *E. coli* (NEB) by chemical transformation, and *TRIM25* CRISPR nuclease constructs were confirmed by sequencing.

**Generation of *TRIM25*-knockout HeLa cells**. *TRIM25* CRISPR nuclease constructs were transfected into HeLa cells by using Effectene transfection reagent (Qiagen) according to the manufacturer's instructions. After 48h transfection, cells were trypsinized and single OFP positive cell was directly sorted into 96 well plate by MoFlo® Cell Sorter (Beckman Coulter) for single cell cloning. Single cell clones were expanded, and then *TRIM25*-knockout HeLa cells were identified by immunoblot analysis with a TRIM25 antibody.

**Analysis of ERG protein stability in VCaP cells.** VCaP cells transfected with siRNA against TRIM25 or a non-targeting control were treated with cycloheximide, or DMSO for 2, 4, 6, or 8 hours starting 72 hours after siRNA transfection. Immunoblot analysis of TRIM25, ERG, and GAPDH was performed, and quantified by densitometry using ImageJ. Band intensities of TRIM25 and ERG were normalized using GAPDH intensity as reference. For quantification three replicate experiments were performed.

**Analysis of ERG ubiquitination in *TRIM25*-knockout HeLa cells.** *TRIM25*-knockout Hela cells grown in DMEM with 10% FBS were co-transfected with ERG-V5 and HA-ubiquitin expression plasmids. Immunoprecipitation with a V5 antibody was performed as described above. For Western blot analysis antibodies against TRIM25, HA, V5, and GAPDH were used.

***In vitro* ubiquitination and deubiquitination**. To obtain purified ERG-V5, ERG-ΔN39-V5 and ERG-ΔN99-V5, transfected HEK293T cells were lysed in lysis buffer (50mM Tris-HCl [pH7.5], 200 mM NaCl, 1% IGEPAL CA-630, 1mM DTT, 5% glycerol and protease inhibitor). Cell

lysates were incubated with mouse anti-V5-antibody (Genscript) for 3 hours at 4°C, followed by immunoprecipitation by the GammaBind G Sepharose beads at 4°C for 1 hour, and targeted proteins were eluted by V5 peptide (Sigma) (50mM Tris-HCl [pH7.5], 100 mM NaCl, 0.1% IGEPAL CA-630, 1mM DTT, 5% glycerol and 500 µg/ml V5 peptide) and dialyzed overnight against dialysis buffer (25mM Tris-HCl [pH7.5], 100 mM NaCl, 1mM DTT). For in vitro ubiquitination assay, purified ERG-V5, ERG-ΔN39-V5 or ERG-ΔN99-V5 (50 µg/ml) and recombinant GST-TRIM25 (20 µg/ml) derived from *E coli* were mixed in a 40µl reaction buffer (50 mM Tris-HCl [pH7.5], 2 mM DTT, 5 mM $MgCl_2$ and 4 mM ATP), containing human recombinant UBE1 (1.0 µg, Boston Biochem), human recombinant UbcH5a (2.0 µg, Boston Biochem) and HA-ubiquitin (10 µg; Boston Biochem). The mixtures were incubated at 32°C for 2 h and subjected to immunoblotting with anti-V5 antibody (Bethyl Laboratories).

To obtain purified USP9X and USP9X catalytically inactive mutant proteins, plasmids encoding Flag-tagged wildtype USP9X and USP9X C1566S-mutant were transfected into HEK293T cells for 48 hours. The cells were lysed in USP9X lysis buffer (50mM HEPES [pH7.5], 300 mM NaCl, 0.2% Triton X-100, 1mM DTT, 5% glycerol) without protease inhibitor, and cell lysates were immunoprecipitated with anti-Flag M2 affinity gel (Sigma) for 3h at 4 °C and eluted with Flag buffer (50mM HEPES [pH7.5], 300 mM NaCl, 1mM DTT, 5% glycerol and 500 µg/ml Flag peptide). Eluted protein was then dialyzed overnight against DUB buffer (50mM HEPES (pH7.5), 100 mM NaCl, 1mM DTT, 5% glycerol). Activity of USP9X-wt-Flag and USP9X-C1566S-Flag was measured using the fluorometric AMC-ubiquitin assay (BostonBiochem) with a fluorometer (Hitachi). For the combined *in vitro* ubiquitination/deubiquitination assay, purified USP9X-wt-Flag, USP9X-C1566S-Flag or mock

were pre-treated with 10 mM DTT for 15 minutes at room temperature, and subsequently incubated in the reaction for ERG ubiquitination by GST-TRIM25 as described above.

**Immunohistochemical staining**

Immunohistochemical staining was performed as previously described (Strand, 2012; Ma, 2010). Briefly, 5 µm sections were deparaffinized, rehydrated and endogenous peroxidases were blocked with 2% hydrogen peroxide in methanol. Sections were then boiled in citrate for antigen retrieval and blocked in 2.5% horse serum for 1 hour. Primary antibodies were incubated at 4°C overnight: ERG (1:100, Biocare medical) and TRIM25 (1:100, Abcam). Biotinylated horse anti-rabbit and horse anti-mouse secondary antibodies (1:500, DAKO Carpentaria) were incubated for 60 minutes at room temperature. Slides where incubated in ABC reagent conjugated with horseradish peroxidase (Vector Laboratories Burlingame) for 30 minutes. Finally, staining was visualized by 3, 3’-diaminobenzidine tetrahydrochloride (DAB substrate) and counterstained with hematoxylin QS (Vector Laboratories Burlingame).

**Immunohistochemical analysis and scoring**

Tumor cells with ERG staining and/or TRIM25 staining were evaluated manually for each tissue core by a reviewer who was blinded to the clinical data. ERG and TRIM25 protein expression levles were registered semi-quantitatively as follows: Staining intensity (0, no staining; 1, weak staining; 2, moderate staining; and 3, intense staining) and the proportion of stained cells (0, no staining; 1, 1-25% staining; 2, 26-50%; 3, 51-75%; and 4, if more than 75% of the tumor cells were positive.

**Analysis of RNA-seq data.** RNA-seq data for prostate adenocarcinoma primary tumor samples was retrieved from TCGA database (N=498). The tags per million (TPM) values for ERG and TRIM25 genes were converted to FPKM values and samples that have ERG expression greater than 25 were selected (N=193). A concordance plot was generated using ERG and TRIM25 expression from the selected 193 ERG-positive tumors.

**Analysis of ERG occupancy at the *TRIM25* gene promoter.** ERG ChIP-seq reads generated for VCaP cells was retrieved from NCBI GEO (GSE14092). SRA files were converted to fastq files and mapped to hg19 using bowtie (v.2.2.5). Bigwig files were generated from uniquely mapped reads using Homer (v. 4.6). For chromatin immunoprecipitation, the chromatin of 10 million VCaP cells were crosslinked with 20 ml of pre-warmed DMEM (37ºC) containing 1% formaldehyde. After 10 minutes of incubation at room temperature 2.2 ml 1.25 M glycine was added, and after 5 minutes of incubation at room temperature the cells were washed twice with ice-cold PBS, scraped off the dish and collected in 5 ml ice-cold PBSpelleted by centrifugation at 700g for 10 minutes. The cell pellet was then lysed in 1 ml ChIP lysis buffer (1% SDS, 10 mM EDTA, 50 mM Tris-HCl [pH 8.1] with protease inhibitors, Roche) and sonicated at 4ºC with a Bioruptor (Diagenode) (30 seconds ON and 30 seconds OFF at highest power for 15 minutes). The sheared chromatin with an average fragment length of ~500 bp was centrifuged at 10,000 g for 10 minutes at 4ºC. 100 μl of the supernatant was used for ChIP or as input. A 1:10 dilution of the solubilized chromatin in ChIP dilution buffer (0.01% SDS, 1.1% Triton X-100, 1.2 mM EDTA, 167 mM NaCl 16.7 mM Tris-HCl [pH 8.1]) was incubated at 4ºC overnight with 6 μg/ml of ERG antibody (Abcam) or IgG (Cell Signaling). Immunoprecipitation was carried out by incubating with 40 μl pre-cleared Protein G Sepharose beads (Amersham Bioscience) for 1 hour

at 4ºC, followed by five washes for 10 minutes with 1ml of the following buffers: Buffer I: 0.1% SDS, 1% Triton X-100, 2 mM EDTA, 20 mM Tris-HCl [pH 8.1], 150 mM NaCl; Buffer II: 0.1% SDS, 1% Triton X-100, 2 mM EDTA, 20 mM Tris-HCl [pH 8.1], 500 mM NaCl; Buffer III: 0.25 M LiCl, 1% NP-40, 1% deoxycholate, 1 mM EDTA, 10 mM Tris-HCl [pH 8.1]; twice with TE buffer [pH 8.0]. Elution from the beads was performed twice with 100 µl ChIP elution buffer (1% SDS, 0.1 M NaHCO3) at room temperature (RT) for 15 minutes. Protein-DNA complexes were de-crosslinked by heating at 65ºC in 192 mM NaCl for 16 hours. DNA fragments were purified using QiaQuick PCR Purification kit (Qiagen) and eluted twice with 30 µl EB buffer according to the manufacturer's protocol after treatment with RNase A and Proteinase K. Quantitative PCR (qPCR) was performed with the oligos 5'-AGTCGCCCATGAGCTG-3' and 5'- ACCCAGGCGTCAGCTTC-3' that amplify a region of the *TRIM25* gene promoter using iTaq™ SYBR® Green Supermix with ROX (Bio-Rad) on a StepOnePlus™ Real-Time PCR Systems (Applied Biosystems). All experiments were performed in triplicate.

**Supplementary Table 1: Ubiquitin ligases identified as ERG binding proteins by mass spectrometry.**

| Gene Name | Accession Number | Unique Peptide Sequences | Coverage (%) | Peptide sequences |
|---|---|---|---|---|
| UBR5 | E7EMW7 | 9 | 4.7 | GSGLLGSQPQPVIPASVIPEELISQAQVVLQGK<br>LLTATNLVTLPNSR<br>NVAIFTAGQESPIILR<br>IVLLSANSIR<br>FAQLALER<br>NSLEDLTAEDFR<br>LLCDSVVLQPYLR<br>AYPAAITILETAQK<br>ATFLGLTNEK |
| TRIM33 | Q9UPN9 | 3 | 3.9 | LLQQQNDITGLSR<br>TPGQINLAQLR<br>SLLQQLENVTK |
| RING1 | Q06587 | 2 | 8 | SLRPDPNFDALISK<br>LHNQQALSSSIEEGLR |
| UBR4 | Q5T4S7 | 6 | 2.2 | NAAQELATLLLSLPAPASVQQQSK<br>TLLPLLLESSTESVAEISSNSLER<br>TLSDVEDQKELASPVSPELR<br>ILGPAESDEFLAR<br>LLQTLPQLR<br>LCSIIYPEENEVTEFFVTLEK |
| RING2 | Q99496 | 7 | 27.1 | FCADCIITALR<br>HNNQQALSHSIEEGLK<br>LALEELR<br>SLRPDPNFDALISK<br>TPQEAITDGLEIVVSPR<br>IYPSRDEYEAHQER<br>TSGNATVDHLSK |
| TRIM25 | Q14258 | 4 | 8.3 | ALLDASETTSTR<br>LPTFGAPEQLVDLK<br>NTVLCNVVEQFLQADLAR<br>VLETFLAK |
| TRIP12 | Q14669 | 3 | 2.3 | IDPLALVQAIER<br>LLDTNPEINQSDSQDSR<br>SSFLASLNPK |
| HUWE1 | Q7Z6Z7 | 7 | 2.6 | AGSSTPGDAPPAVAEVQGR<br>LGSSGLGSASSIQAAVR<br>LLGPSAAADILQLSSSLPLQSR<br>SSDPLGDTASNLGSAVDELMR<br>TVLNQILR<br>LLSLISIALPENK<br>VLLSPDYLPAMR |
| CHIP | Q9UNE7 | 2 | 11.7 | SPLTQEQLIPNLAMK<br>LNFGDDIPSALR |
| UHRF1 | Q96T88 | 8 | 11.7 | ALALNCFAPINDQEGAEAK<br>AQVFSCPACR<br>ELYANVVLGDDSLNDCR<br>LNDTIQLLVR<br>NDASEVVLAGER<br>TKVEPYSLTAQQSSLIR<br>VEPYSLTAQQSSLIR<br>VNEYVDAR |
| HERC2 | O95714 | 2 | 0.5 | SAQGTSAPLLPLLQR<br>EDVESQNK |

## Supplementary Table 2: Sequences for all primers and siRNAs.

| | | |
|---|---|---|
| **siRNAs** | | |
| siTRIM25-1 | 5'-CGGAACAGUUAGUGGAUUU-3' | |
| siTRIM25-2 | 5'-CAACAAGAAUACACGGAAA-3' | |
| siERG-1 | 5'-GAUCCUACGCUAUGGAGUA-3' | |
| siERG-2 | 5'-GGACAGACUUCCAAGAUGA-3' | |
| | | |
| **Cloning Primers** | | |
| TRIM25_CDS_F | 5'-CCGCTCGAGAGCGCCATGGCAGAGCTGTGC-3' | Xho I |
| TRIM25_CDS_R | 5'-ATAAGAATGCGGCCGCTCACTTGTCGTCATCGTCTTTG-3' | Not I |
| TRIM25_GST_F | 5'-CCGGAATTCCCATGGCAGAGCTGTGC-3' | EcoR I |
| TRIM25_GST_R | 5'-CCGCTCGAGTCACTTGGGGGAGCAGATGGAG-3' | Xho I |
| TRIM25-ΔRing-F | 5'-CCGCTCGAGATGTACCAGGCGCGACCGCAGCTG-3' | |
| TRIM25-ΔCCD-F | 5'-GCATCTGCCACATCTGCCTGGAGCTCCTGGAGTATTACATTAAAGTC-3' | |
| TRIM25-ΔCCD-R | 5'-GACTTTAATGTAATACTCCAGGAGCTCCAGGCAGATGTGGCAGATGC-3' | |
| TRIM25-ΔSPRY-R | 5'-GCTCTAGATTTAATGTAATACTCCAGGAG-3' | |
| ERG_GST_F | 5'-TCCCCCGGGGGAATGATTCAGACTGTCCCGGAC-3' | Xma I |
| ERG_GST_His_R | 5'-CCGCTCGAGCTATCAGTGGTGGTGGTGGTGGTGGTAGTAAGTGCCCAGATGAG-3' | Xho I |
| ERG_TnT_F | 5'-CCGCTCGAGATGATTCAGACTGTCCCGGAC-3' | Xho I |
| ERG_TnT_R | 5'-TCCCCCGGGGTAGTAAGTGCCCAGATGAG-3' | Xma I |
| ERG_ΔNAD_F | 5'-CCGCTCGAGATGAACTACGGCAGCTACATG-3' | Xho I |
| ERG_ΔNAD_PNT_F | 5'-CCGCTCGAGATGCATGCTAGAAACACAGG-3' | Xho I |
| ERG_N951_R | 5'-GCTCTAGACTGGCCACTGCCTGGATTTGCAAG-3' | Xba I |
| ERG_ΔE_F | 5'-CTTGCAAATCCAGGCAGTGGCCAGTTCCACGGGATCGCCCAGGCCCTC-3' | |
| ERG_ΔE_R | 5'-GAGGGCCTGGGCGATCCCGTGGAACTGGCCACTGCCTGGATTTGCAAG-3' | |
| | | |
| **esiRNA primers** | | |
| CHIP_F | 5'-TCACTATAGGGAGACTGAAGGGCAAGGAGGAGAAG-3' | |
| CHIP_R | 5'-TCACTATAGGGAGAGCTCCTCAATGCTGTTCCAG-3' | |
| HERC2_F | 5'-TCACTATAGGGAGAGACAGCAGCGACGATTCAG-3' | |
| HERC2_R | 5'-TCACTATAGGGAGACAAGGCTTTATTCATTTTGGGG-3' | |
| HUWE1_F | 5'-TCACTATAGGGAGAGCACAGAAGTTCCTTCGCTTTG-3' | |
| HUWE1_R | 5'-TCACTATAGGGAGACACCAGGTGAGGTACGGAAC-3' | |
| RING1_F | 5'-TCACTATAGGGAGAGAAGCCATAATGGATGGCAC-3' | |
| RING1_R | 5'-TCACTATAGGGAGACCCACTCATCGTTGTGGTCT-3' | |
| RING2_F | 5'-TCACTATAGGGAGAGTCTCAGGCTGTGCAGACAA-3' | |
| RING2_R | 5'-TCACTATAGGGAGACTTCAGTCCTTCCTCAATGCTG-3' | |
| TRIM25_F | 5'-TCACTATAGGGAGAGTCGAGAGCACTGGATGATG-3' | |
| TRIM25_R | 5'-TCACTATAGGGAGACGATGCACTGCTTCAGCTCGT-3' | |
| TRIM33_F | 5'-TCACTATAGGGAGAGAAGATGTCTCAGAGTCTGTTGGA-3' | |
| TRIM33_R | 5'-TCACTATAGGGAGACGGGAAAGGCCTGTGATGTC-3' | |
| TRIP12_F | 5'-TCACTATAGGGAGAGAAGGGTGGACCTGTCAAGA-3' | |
| TRIP12_R | 5'-TCACTATAGGGAGACACACTCCATCGTGCCATAA-3' | |
| UBR4_F | 5'-TCACTATAGGGAGACTGGAGGACATGACCACAGG-3' | |
| UBR4_R | 5'-TCACTATAGGGAGAGCTCTCCCTCATTCGTGGTA-3' | |
| UBR5_F | 5'-TCACTATAGGGAGACCTGGCAAGAACACAGAAGCA-3' | |
| UBR5_R | 5'-TCACTATAGGGAGAGTCATTGCATGTAAGGGCTGT-3' | |
| UHRF1_F | 5'-TCACTATAGGGAGAGCTCTCCGACACCGACTC-3' | |
| UHRF1_R | 5'-TCACTATAGGGAGACGGGTTGTCGGGGTTGTAGTT-3' | |
| | | |
| **Realtime PCR primers** | | |
| ERG_F | 5'-CGCAGAGTTATCGTGCCAGCAGAT-3' | |
| ERG_R | 5'-CCATATTCTTTCACCGCCCACTCC-3' | |
| TRIM25_F | 5'-GTTCGCGACCTGTTGCGCCG-3' | |
| TRIM25_R | 5'-TGTGCCTCAGGGTGGCCTCCAG-3' | |
| GAPDH_F | 5'-CGACCACTTTGTCAAGCTCA-3' | |
| GAPDH_R | 5'-AGGGGAGATTCAGTGTGGTG-3' | |

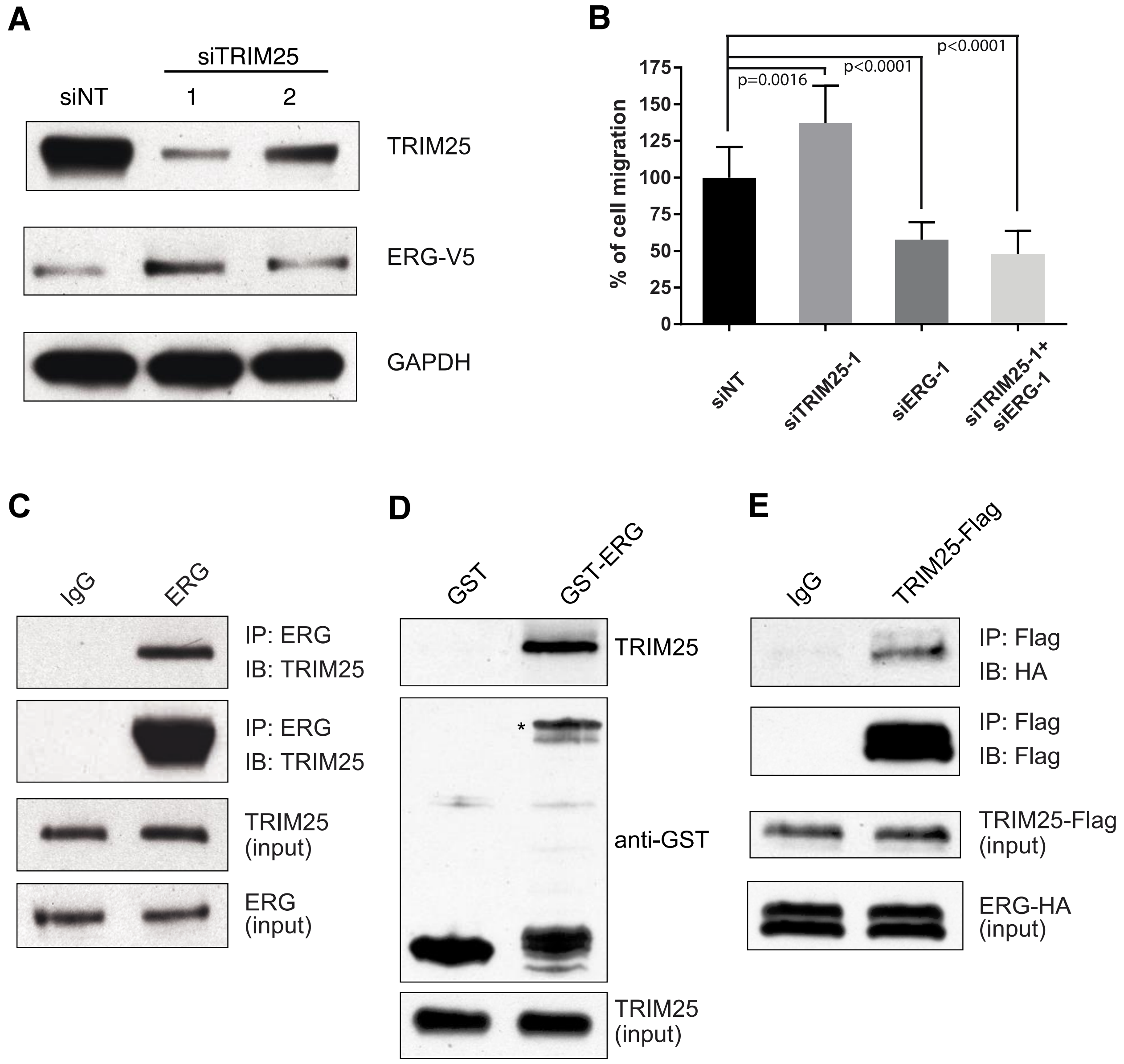


**Supplementary Figure 1: TRIM25 affect ERG protein levels and function and physically interacts with ERG**.

**A.** TRIM25 knockdown increases ERG-V5 protein levels in 22Rv1 cells. TRIM25 expression was knocked down with two independent siRNAs, and a non-targeting siRNA (siNT) was used as control. ERG protein levels were assayed 72 hours after knockdown. **B**. TRIM25 knockdown

increases VCaP cell migration, which is reduced by ERG knockdown. Cells were transfected with siRNAs for 48 hours, and then subjected to the Transwell assay (n=8, *p* values - *t* test, error bars represent s.d.). **C**. Co-immunoprecipitation of TRIM25 with ERG in VCaP cells. VCaP cells were used for immunoprecipitation of ERG with an ERG antibody. Immunoblotting was performed with antibodies against ERG and TRIM25. **D**. Pulldown of TRIM25 with GST-ERG from VCaP cell extract. GST-ERG was expressed in *E. coli* and used to pull down proteins from whole VCaP cell extract. Immunoblotting was performed with antibodies against GST and TRIM25. **D**. Co-immunoprecipitation of ERG-HA with TRIM25-Flag. ERG-HA and TRIM25-Flag were co-expressed in HEK293T cells. Immunoprecipitation was performed with an antibody against Flag. Immunoblotting was performed with antibodies against HA and Flag. For all experiments the immunoblots shown for input represents 1% input. IgG was used as a negative control for immunoprecipitation the co-immunoprecipitation experiments, and in the pulldown experiment GST was used as a negative control.

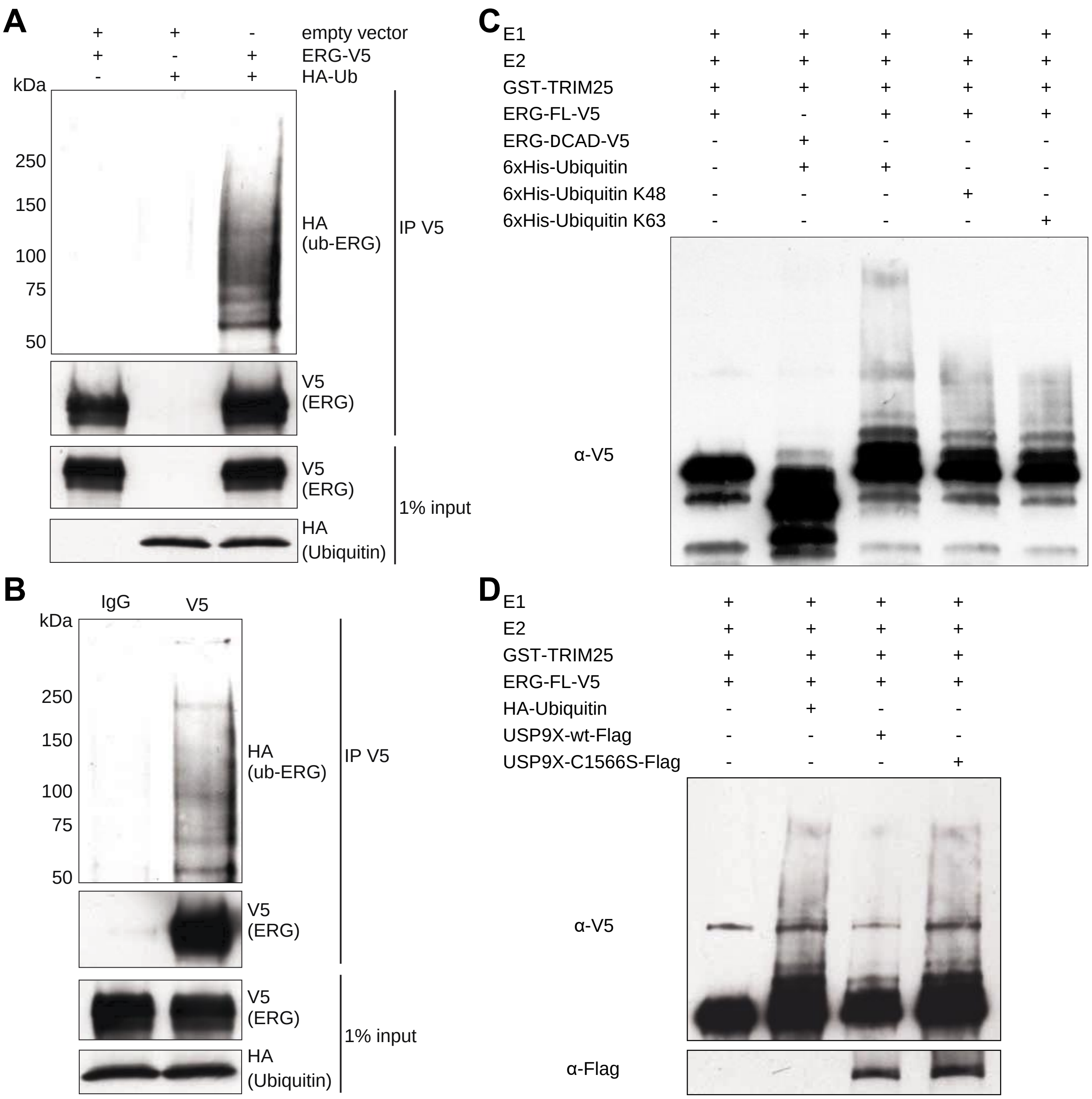


**Supplementary Figure 2: ERG ubiquitination and deubiquitination *in vitro* assays with TRIM25 and USP9X.**

**A., B.** Specificity analysis for the ERG ubiquitination assay. HeLa cells were co-transfected with the empty pCMV-TnT expression vector, the ERG-V5, or HA-ubiquitin expression constructs; or the ERG-V5 and HA-ubiquitin expression constructs (**A**). Immunoprecipitation was performed with a V5 antibody. Western blot analysis for immunoprecipitated proteins and input

was performed with antibodies against V5 and HA. HeLa cells were co-transfected with the ERG-V5 and HA-ubiquitin expression constructs. Immunoprecipitation was performed with an antibody directed against V5 or IgG (**B**). Western blot analysis for immunoprecipitated proteins and input was performed with an antibody against V5 and HA. **C**. TRIM25 mediates K48- and K63-linked ubiquitination of ERG *in vitro*. ERG-V5 variants (full length – ERG-FL-V5, CAD deletion variant – ERG-ΔCAD-V5) were ectopically expressed in HEK293T cells, and immunoprecipitated for an *in vitro* ubiquitination assay using GST-TRIM25 (expressed in *E. coli*), E1 and E2 conjugating enzymes, 6xHis-Ubiquitin, 6xHis-Ubiquitin K48, 6xHis-Ubiquitin K63 (all purchased from Boston Biochem). Immunoblotting was performed with V5 antibodies. **D.** TRIM25-mediated ubiquitination is reversed by USP9X. The assay was performed as described for (**C**) in absence or presence of catalytically active USP9X (USP9X-wt-Flag) or catalytically inactive USP9X (USP9X-C1566S-Flag).

## SUPPLEMENTAL REFERENCES


Kittler, R., Heninger, A.K., Franke, K., Habermann, B., and Buchholz, F. (2005). Production of endoribonuclease-prepared short interfering RNAs for gene silencing in mammalian cells. Nat Methods *2*, 779-784.

Kittler, R., Putz, G., Pelletier, L., Poser, I., Heninger, A.K., Drechsel, D., Fischer, S., Konstantinova, I., Habermann, B., Grabner, H., *et al.* (2004). An endoribonuclease-prepared siRNA screen in human cells identifies genes essential for cell division. Nature *432*, 1036-1040.

Kittler, R., Surendranath, V., Heninger, A.K., Slabicki, M., Theis, M., Putz, G., Franke, K., Caldarelli, A., Grabner, H., Kozak, K., *et al.* (2007). Genome-wide resources of endoribonuclease-prepared short interfering RNAs for specific loss-of-function studies. Nat Methods *4*, 337-344.
